# Hypoglossal nerve stimulation for treatment of obstructive sleep apnea (OSA): a primer for oral and maxillofacial surgeons

**DOI:** 10.1186/s40902-017-0126-0

**Published:** 2017-09-25

**Authors:** Sung ok Hong, Yu-Feng Chen, Junho Jung, Yong-Dae Kwon, Stanley Yung Chuan Liu

**Affiliations:** 1 Department of Dentistry (Oral and Maxillofacial Surgery), Catholic Kwandong University School of Medicine, International St. Mary’s Hospital, Incheon, South Korea; 2 Stanford University School of Medicine, Stanford, CA USA; 3 Department of Oral and Maxillofacial Surgery, Kaohsiung Medical University Hospital, Kaohsiung, Taiwan; 4 Department of Oral and Maxillofacial Surgery, Kyung Hee University Dental Hospital, Seoul, Republic of Korea; 5 Department of Oral and Maxillofacial Surgery, Center for Refractory Jawbone Diseases, School of Dentistry, Kyung Hee University, Seoul, Republic of Korea; 6 Department of Otolaryngology, Stanford University School of Medicine, Stanford, CA USA

**Keywords:** Hypoglossal nerve, Upper airway stimulation, Obstructive sleep apnea, Snoring, Sleep endoscopy, OSA surgical treatment, Oral and maxillofacial surgeon

## Abstract

The prevalence of obstructive sleep apnea (OSA) is estimated to be 1–5% of the adult population world-wide, and in Korea, it is reported at 4.5% of men and 3.2% of women (Age 40 to 69 years old). Active treatment of OSA is associated with decrease in insulin resistance, cardiovascular disease, psychosocial problems, and mortality. Surgical treatment of OSA has evolved in the era of neuromodulation with the advent of hypoglossal nerve stimulation (HGNS). We share this review of HGNS with our maxillofacial surgical colleagues to expand the scope of surgical care for OSA.

## Introduction

### OSA prevalence

The prevalence of obstructive sleep apnea (OSA) is estimated to be 2–5% of the adult population [1]. In Korea, OSA is reported in 4.5% of men and 3.2% of women between the ages of 40 to 69 years [2]. Despite a lower prevalence of obesity as compared to Western countries, the prevalence is similar in Korea. Active treatment of OSA decreases the incidence of insulin resistance, cardiovascular disease, psychosocial problems, and mortality [3–6].

### OSA treatment

Non-surgical treatments for OSA include weight loss, behavioral modifications, mandibular advancement device (MAD), and continuous positive airway pressure (CPAP). CPAP is considered as first-line treatment for OSA with well documented efficacy and morbidity. As a treatment modality, its adherence rate ranges from 39 to 50% [7–9]. Surgical options include soft tissue and skeletal reconstruction of the upper airway. Soft tissue procedures include septoplasty, various forms of uvulopalatopharyngoplasty, tongue base reduction, and hyoid suspension. Common skeletal procedures include genioglossus advancement, maxillary expansion, and maxillomandibular advancement (MMA). Except for tracheostomy and MMA, anatomically modifying surgeries of the upper airway report success rates ranging from 20% to 60% [10]. Besides tracheostomy, MMA is the most effective surgical treatment with success rates as high as 85.5% [11] Neurostimulation for stability of the upper airway during sleep was introduced as an option that may be less invasive and more effective in the well-selected patient [12].

## Review

### Upper airway stimulation (hypoglossal nerve stimulation)

#### History and evolution of the HGNS concept

Animal studies have confirmed that the genioglossus muscle is a key protrusion muscle, as opposed to the styloglossus and hyoglossus muscles which retract the tongue [13]. In 1989, Miki et al. found a relationship between the hypoglossal nerve and upper airway resistance during stimulation in six canines [14]. In 1992, Schwarz et al. reported a correlation of V1max stimulation and decrease in critical closing pressure (Pcrit) in 18 decerebrate felines. Yoo et al. suggested that multi-contact nerve electrodes can be effective in achieving upper airway dilation and patency by selective activation of various branches of the hypoglossal nerve in eight beagles [15]. Oliven et al. reported on the effect of airway modulation by selectively stimulating protrusor and retrusor muscles in anesthetized canines [16]. The study confirmed that selective genioglossus muscle stimulation significantly stabilized the airway while stimulation of the styloglossus and hyoglossus muscles collapsed the airway.

The first attempt to improve upper airway patency in humans via neurostimulation was performed by Guilleminault et al. [17]. Transcutaneous submental and intraoral electrical stimulation of the upper airway muscles was attempted with limited success. Preliminary successful human studies were first reported in 1996 by Schwartz et al. [18]. By intramuscular stimulation of the lingual muscle in nine participants, the frequency of airway collapse decreased without causing sleep arousal. A pilot study in 2001 proposed that unilateral electrical stimulation of the hypoglossal nerve was a feasible and potential therapeutic option for OSA [19]. Efficacy of OSA treatment was shown in seven of the eight hypoglossal nerve stimulation (HGNS) implanted patients (Inspire Medical systems, Maple Grove, MN). For the 6-month continuation of the study, the results were consistent; they were not successful in the long term due to technical defects that led to device dysfunction [20].

#### HGNS devices and device concepts

Following the published technical limitations of the human pilot study in 2001, several investigators and medical device companies focused on improvements over a decade. In 2011, three clinical trials were sponsored by different and independent firms: Apnex (St. Paul, MN, USA), Inspire (Maple Grove, MN, USA), and ImThera Medical (San Diego, CA, USA).

Apnex and Inspire are based on unilateral inspiratory stimulation to the medial branch of the hypoglossal nerve. The two devices differed in inspiration sensors; Apnex was a double impedance sensor positioned over the lower ribs of both hemithoraces while Inspire was an effort sensor placed between the intercostal muscles.

Eastwood et al. [21] were the first to report results from the Apnex trial. This clinical trial was performed in Australia enrolling 37 patients and ultimately implanting 21. Inclusion criteria consisted of patients with AHI between 20 and 100 events per hour (at least 80% hypopnea), BMI < 40 (kg/m2), and age between 21 and 70 years. The 6-month follow-up yielded successful results with more than 50% reduction in the AHI. But the larger scale phase 2–3 clinical trial did not deliver anticipated results with mean AHI decrease from 45 to 25 [22]. Surgically unsuccessful results led to cessation of device development.

Van de Heyning et al. [23] subsequently published the results from the 6-month clinical trial with the Inspire II device. It was a two-part study with the first consisting of a wide inclusion and exclusion criteria. The 20 patients enrolled in the study had BMI < 35 (kg/m2) and AHI > 25 events per hour. Fourteen patients showed the predicted outcome, and six patients showed greater than 50% reduced AHI that was also less than 20 events per hour after 6 months. The second part narrowed the criteria based on the findings from part one and included drug-induced sleep endoscopy (DISE) as a diagnostic modality. The inclusion criteria were BMI less than 32 (kg/m2), AHI between 20 and 50 events per hour, and absence of complete concentric collapse (CCC) of the velum as seen during DISE. Seven of the eight implanted patients assessed at 6 months post operatively showed successful results.

Succeeding the Inspire II trial, a larger multicenter 1-year phase 2–3 trial (STAR trial) was completed in 2014 by Strollo et al. [12]. One hundred twenty-six patients were enrolled with inclusion criteria consisting of CPAP non-adherence, BMI less than 32 (kg/m2), AHI between 20 to 50 events per hour, absence of significant positional or central apneas, and absence of CCC on DISE at the velum. Sixty-six percent of the patients had achieved surgical success. The device was then FDA approved.

Mwenge et al. [24] performed a 1-year clinical study treating 14 OSA patients with the ImThera Medical device. Stimulation was delivered at both inspiration and expiration and thereby excluding the need of an inspiratory sensor. The hypoglossal nerve electrode was cuffed on the main trunk and targeted all muscles in the hemilateral tongue. Inclusion criteria consisted of BMI between 25 and 40 (kg/m2), AHI below 20 events per hour, Mallampati score 1–3, and palatine tonsils grade 0–2. Ten of the 13 implanted patients achieved surgical success with mean AHI decrease from 45.2 to 21 events per hour. Currently, a larger phase 2–3 trial is underway.

#### Surgical technique

The HGNS system (Inspire, Maple Grove, MN, USA) is comprised of three parts: stimulation cuff electrode, pleural pressure sensing lead, and implantable pulse generator (IPG).

##### Simulation cuff electrode

**Fig. 1:**
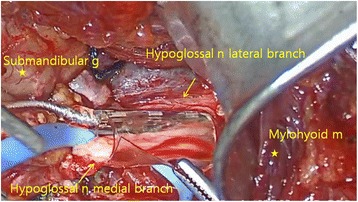
Cuff electrodes encircling the medial branch of hypoglossal nerve with three stimulation nodes. Note the lateral branch is not included in the cuff. (*n* nerve, *m* muscle, *g* gland)

The stimulation cuff electrode is wrapped around the medial branches of the right hypoglossal nerve and protrudes and stiffens the tongue to mitigate airway collapse. First, a 5-cm horizontal incision is made 1 fingerbreadth below the right mandible border with the submandibular gland as the posterior boundary extending anteriorly towards the midline. The digastric tendon is identified and retracted inferiorly while the submandibular gland is lifted postero-superiorly. The posterior edge of the mylohyoid muscle is retracted anteriorly to visualize the space where the main trunk of the hypoglossal nerve is usually positioned and branches. The vena comitans overlying the nerve can be ligated for better access of the hypoglossal nerve branches. The functional break point is located between the medial and lateral branches of the main hypoglossal nerve trunk. Using a nerve integrity monitoring system (NIM, Medtronic Xomed, Jacksonville, Fl), [10] the medial branches of the hypoglossal nerve are confirmed and the cuff selectively wraps around these branches (Fig. 1).

##### Pleural pressure sensing lead

**Fig. 2:**
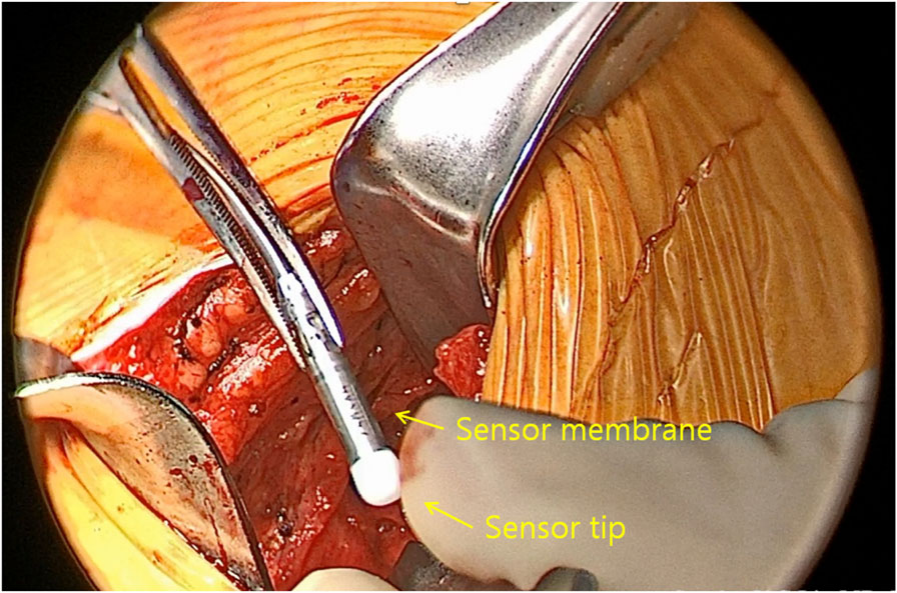
Pleural pressure sensing lead is placed with the ventilatory sensor facing the pleura

The pleural pressure sensing lead detects ventilator effect and sends this information to the IPG which in turn activates the hypoglossal nerve stimulation cuff. The respiratory effort-sensing lead coordinates stimulation during the most vulnerable part of the respiratory cycle (end expiratory phase). Stimulation is given between end-expiration through the inspiratory period to minimize neuromuscular fatigue [25]. A horizontal incision is made at the right fourth or fifth intercostal space lateral to the nipple line. Dissection is carried out to the upper border of the underlying rib, and a pocket is tunneled postero-anteriorly between the external and internal intercostal muscle layers where the sensing lead faces the pleura (Fig. 2).

##### Implantable pulse generator

The IPG connects both the hypoglossal nerve stimulation cuff and pleural sensing electrode. It receives the information from the pleural sensing electrode and generates a pulse that is transferred to the stimulation cuff. The IPG pocket is created 2 to 5 cm inferior to the right clavicle and medial to the delto-pectoral groove. The pocket is deep to the subcutaneous fat and superficial to the pectoralis major muscle fascia. Inferiorly, the pocket is extended by tunneling subcutaneously to the pleural sensing electrode. Superiorly, the pocket is extended from the clavicle to the hypoglossal nerve stimulation cuff by tunneling at the subplatysmal level.

**Fig. 3:**
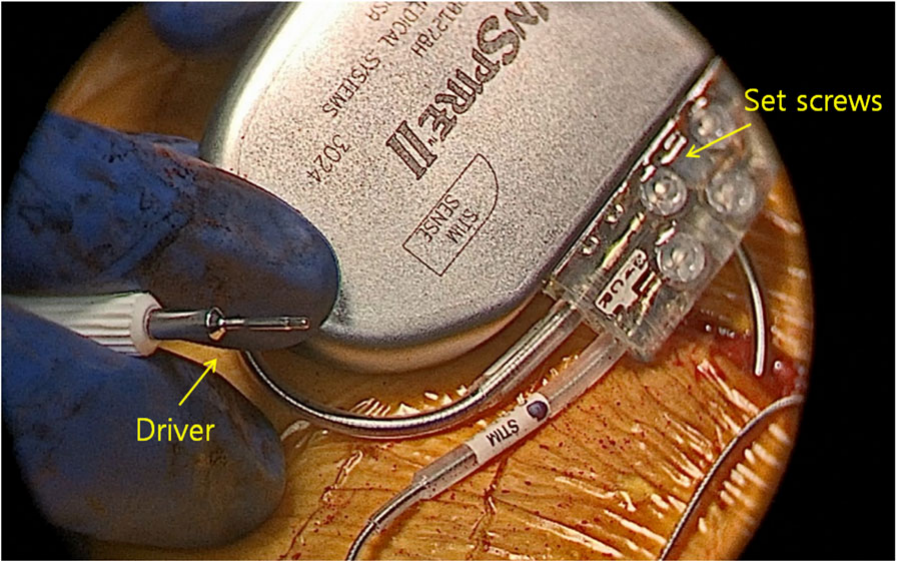
Implantable pulse generator (IPG) has two 3.2 mm low profile connector ports (STIM port, SENSE port) which house the stimulation and pleural pressure sensing lead connectors. The lead connectors are secured with set screws using a driver

After HGNS systems are implanted, the IPG is tested to confirm tongue protrusion prior to closure (Fig. 3).

#### Advantages of HGNS

Even though CPAP is a treatment for OSA that has well-documented efficacy and low morbidity, the adherence rate is low [7]. In patients with CPAP intolerance, HGNS can be an effective alternative. Daily use of the device at 12 months was 86% and daily use at 18 months was 84% [26]. The advantage of HGNS being a titratable therapy like CPAP or MAD is especially beneficial as OSA is a chronic condition that needs monitoring and re-evaluation due to aging, weight gain, and decrease in elasticity of the soft tissues.

Another advantage of HGNS is that as a one-time procedure it improves airway collapsibility at multiple levels. DISE and fluoroscopy studies have shown retrolingual and retropalatal space enlargement with HGNS [27, 28]. HGNS directly opens the hypopharyngeal airway with tongue protrusion, but it also impacts the retropalatal airway. Its mechanism restores muscle tone to avoid upper airway collapse during sleep.

#### Complications of HGNS

Strollo et al. announced the overall rate of serious adverse effects to be less than 2% [12] with no serious IPG device infection requiring explantation and no permanent hypoglossal nerve damage. There is also less postoperative discomfort as compared to other soft tissue and skeletal operations [25]. Discomfort in the IPG position, tongue stiffness, tongue abrasion, transient ipsilateral tongue paresis, and post-operative swelling were the reported side effects. Most were minor and can be mitigated by meticulous surgical techniques, adjustment of stimulation parameters, dental adjustments, and the use of mouth guards.

#### Limitations of HGNS

Woodson et al. reported the 36-month postoperative outcome data of the STAR trial patients [29]. Continued follow-up supporting the use of HGNS is warranted. Another limitation is the narrow selection criteria. Patients over BMI 32 kg/m2 are excluded from HGNS as success rates become inconsistent. Nevertheless, the prevalence of obesity is high and continues to increase [30]. The high cost of the HGNS implant is another limitation to wide application. Another drawback of HGNS is the current size of the IPG and MRI incompatibility.

## Conclusions

### Role of oral and maxillofacial surgeons

Beyond soft tissue and skeletal reconstruction of the upper airway, oral and maxillofacial surgeons should be familiar with neuromodulation of the upper airway and its latest outcomes and technique updates. With unpredictability of surgical results from soft tissue surgery and the reluctance of many patients to undergo major facial skeletal changes, HGNS is a viable option that is needed in the scope of sleep apnea surgical care. Evaluation of dynamic airway collapse patterns using DISE is an important prerequisite.

### Conclusion

Currently, only one device is FDA approved for HGNS. The inclusion criteria are failure of CPAP adherence, BMI less than 32 kg/m2, AHI between 20 to 50 events per hour, absence of CCC seen on DISE at the velum, and absence of significant positional or central apneas. Stanford’s sleep surgery team with maxillofacial surgeons Riley and Liu has reported the first post-MMA relapse followed by successful treatment of OSA via HGNS [31]. Familiarity with HGNS as an alternative to upper airway reconstruction augments the maxillofacial surgeon’s comprehensive care for patients with OSA.
